# ﻿Taxonomic notes on the genus *Aprostocetus* Westwood (Hymenoptera, Eulophidae) from China, with the description of a new species

**DOI:** 10.3897/zookeys.1253.163155

**Published:** 2025-09-26

**Authors:** Wen-Jian Li, Rui Liu, Zhi-Peng Chen, Guo-Hao Zu, Cheng-De Li

**Affiliations:** 1 Jiangsu Provincial Key Laboratory of Coastal Wetland Bioresources and Environmental Protection, School of Wetland, Yancheng Teachers University, Yancheng, 224007, China Yancheng Teachers University Yancheng China; 2 College of Horticulture and Landscape, Tianjin Agricultural University, Tianjin 300392, China Tianjin Agricultural University Tianjin China; 3 School of Forestry, Northeast Forestry University, Harbin, 150040, China Northeast Forestry University Harbin China

**Keywords:** Chalcidoidea, parasitoids, taxonomy, Tetrastichinae

## Abstract

Fourteen species of *Aprostocetus* Westwood from China are reviewed, including one new species, *A. maculosus***sp. nov.**, and new country records for four species: *A. viridinitens* Graham, 1987, *A. occidentalis* Graham, 1987, *A. ligus* (Walker, 1839) and *A. percaudatus* (Silvestri, 1920). New distributional data for *A. caudatus* Westwood, 1833, *A. epicharmus* (Walker, 1839), *A. gratus* (Giraud, 1863), *A. minimus* (Ratzeburg, 1848), *A. microcosmus* (Girault, 1917), *A. pinus* Li & Xu, 2014, *A. nigroplaque* Yang & Cao, 2015, *A. crino* (Walker, 1838), and *A. mandanis* (Walker, 1839) are provided. Keys to Chinese subgenera of the genus *Aprostocetus* and Chinese species of the subgenera *Aprostocetus* and *Ootetrastichus* are given based on Chinese material.

## ﻿Introduction

The genus *Aprostocetus* Westwood (Eulophidae: Tetrastichinae) was established by Westwood (1833) with *Aprostocetus caudatus* Westwood as the type species. *Aprostocetus* is one of the most species-rich genera within the family Eulophidae, containing 816 described valid species and exhibiting a broad global distribution across the world’s major zoogeographic regions (UCD Community 2023). Graham (1987) divided *Aprostocetus* into five subgenera: *Aprostocetus*, *Chrysotetrastichus* Kostjukov, *Tetrastichodes* Ashmead, *Ootetrastichus* Perkins and *Coriophagus* Graham. LaSalle (1994) introduced a new subgenus, *Quercastichus* LaSalle. Although *Aprostocetus* comprises 816 species in six subgenera worldwide, only 43 species in three subgenera (*Aprostocetus*, *Tetrastichodes*, *Ootetrastichus*) are currently known from China.

*Aprostocetus* are widely distributed and can be recognized by the following combination of characteristics: malar sulcus present; antennal funicle with three or four segments, clava with two in *A. gratus* (Fig. 27) or three segments; mid lobe of mesoscutum usually with one row of adnotaular seta, in a few species with two to three rows; scutellum mostly with submedian grooves and sublateral grooves, rarely absent; propodeum with median carina present, plicae absent, paraspiracular carina usually absent, rarely present, spiracle often partly covered by a raised lobe of the callus; submarginal vein with two or more dorsal setae; postmarginal vein absent or at most 0.5 × length of stigmal vein; one cercal seta often much longer than the next longest, often sinuate/kinked medially; ovipositor sheaths usually at least slightly projecting, rarely not projecting; mesopleuron with precoxal suture, mesosternum flat in front of the trochantinal lobes (Graham 1987). Among the numerous genera in the subfamily Tetrastichinae, *Aprostocetus* does not have any unique characteristics, except some species (e.g. *A. percaudatus*) with extremely elongated ovipositor sheaths. In practice, if a tetrastichine species does not have the unique characteristics of other genera, it is usually considered to belong to *Aprostocetus*. *Aprostocetus*, especially the subgenus Ootetrastichus, is often most similar to *Quadrastichus* in having a reduced number of adnotaular setae and a slender body; however, *Quadrastichus* normally has only one dorsal seta on the stigmal vein and is at most only weakly metallic compared to many *Aprostocetus*. Some *Aprostocetus* species are quite similar to *Baryscapus* in having more than one row of adnotaular setae; however, *Baryscapus* can be distinguished by the metallic tints on the tegulae and the fully exposed rim of the propodeal spiracle. Also, a few species are similar to *Neotrichoporoides* in having a long thorax and a sclerotized, metallic body; however, *Neotrichoporoides* has a longer marginal vein and propodeum than *Aprostocetus*.

Species of *Aprostocetus* are generally primary parasitoids of hosts inhabiting plant galls, such as Cecidomyiidae (Diptera) and Cynipoidea (Hymenoptera) (Graham 1987; Bouček 1988; LaSalle 1994). Other hosts include many families of Hemiptera, Coleoptera, Lepidoptera, Blattodia, Orthoptera, and Neuroptera (Graham 1987; LaSalle 1994; de Medeiros 2009). Furthermore, some species of *Aprostocetus* infest plants, resulting in the formation of galls (Beardsley and Perreira 2000; Li et al. 2014). An unidentified *Aprostocetus* species was even reported as a larval predator of a nematode species (Berg 1990).

In this paper we add five more species, including one new species and four new country records to the Chinese fauna. Also, keys to the Chinese subgenera of the genus *Aprostocetus* and Chinese species of the subgenera *Aprostocetus* and *Ootetrastichus* are given based on females.

## ﻿Materials and methods

Specimens were collected by sweeping vegetation and yellow-pan trapping. Specimens were dissected and mounted dorsally in Canada balsam following the method of Noyes (1982) or glued to triangular cards. Photographs were taken with a Leica DFC500 digital camera attached to a Lecia DM4000B compound microscope. Slide-mounted specimen measurements were made using an eye-piece reticle with an Olympus CX21 optical microscope. Card-mounted specimens were measured in absolute ethanol prior to mounting using an eye-piece reticle with a Motic SMZ168-B stereo microscope. In the descriptions below, measurements or ratios refer to that of the holotype. Terminology follows Graham (1987) and Gibson et al. (1997). The following abbreviations are used:

**C1–3** clavomeres 1–3

**F1–4** flagellomeres 1–4

**POL** minimum distance between lateral ocelli

**OOL** minimum distance between lateral ocellus and eye margin

**OD** longest diameter of a lateral ocellus

**MV** marginal vein

**STV** stigmal vein

**SMV** submarginal vein

**PMV** postmarginal vein

Each of the specimens studied in this paper are deposited in the insect collections of Yancheng Teachers University (**YCTU**), Yancheng, Jiangsu, China.

## ﻿Results

### ﻿Key to subgenera of *Aprostocetus* Girault from China (females)

**Table:** 

1	Mid lobe of mesoscutum with numerous suberect setae; meso- and metabasitarsus 1.5 × as long as the corresponding second tarsomere	***Tetrastichodes* Ashmead**
–	Mid lobe of mesoscutum normally with 1 row of adnotaular setae on each side, occasionally 2 or 3 rows of adnotaular setae; meso- and metabasitarsus shorter than or as long as the corresponding second tarsomere	**2**
2	Forewing SMV usually with 2 dorsal setae, rarely 3–5 dorsal setae, speculum small (e.g. Fig. 36), sometimes absent (e.g. Figs 38, 40); propodeal spiracles small with the whole rim exposed (e.g. Fig. 41)	***Ootetrastichus* Perkins**
–	Forewing SMV usually with > 2 dorsal setae, rarely 2 dorsal setae, speculum moderated-sized (e.g. Fig. 8) or large (e.g. Fig. 30); propodeal spiracles moderated-sized with the rim partly covered by a raised lobe of the callus (e.g. Figs 11, 14, 31)	***Aprostocetus* Girault**

### ﻿Key to species of the subgenus Aprostocetus Girault from China (females)

**Table:** 

1	Mid lobe of mesoscutum with 2 or 3 rows of adnotaular setae on each side	**2**
–	Mid lobe of mesoscutum with 1 row of adnotaular setae on each side	**3**
2	Mid lobe of mesoscutum dull, with distinctly raised reticulation; body black without yellow markings (Fig. 30)	***A. microcosmus* (Girault)**
–	Mid lobe of mesoscutum glossy, with shallow reticulation; body yellow with recognizable black markings (Figs 1, 2)	***A. maculosus* sp. nov.**
3	Forewing with MV short, 1.8–2.0 × as long as STV	**4**
–	Forewing with MV moderately long, > 2.0 × as long as STV	**5**
4	Antenna with F1 2.2 × as long as broad	***A. orgyiae* Yang & Yao**
–	Antenna with F1 1.6 × as long as broad (Fig. 34)	***A. nigroplaque* Yang & Cao**
5	Forewing with MV > 4.5 × as long as STV	**6**
–	Forewing with MV 2.0–4.5 × as long as STV	**11**
6	Gaster lanceolate, last tergite length at most slightly greater than width; ovipositor sheaths slightly exserted (e.g. Fig. 7)	**7**
–	Gaster elongated-lanceolate, last tergite length distinctly greater than width; ovipositor sheaths distinctly exserted (e.g. Fig. 15)	**8**
7	Pronotum conical, 0.4 × as long as mesoscutum; body with strong metallic tints	***A. foraminifer* Graham**
–	Pronotum short, < 0.4 × as long as mesoscutum; body without metallic tints	***A. nubigenus* Graham**
8	Antenna with F1 4.5 × as long as broad	***A. dendroctoni* Yang**
–	Antenna with F1 1.6–2.5 × as long as broad	**9**
9	Ovipositor sheaths plus postcercale 1.2 × as long as metatibia; antenna with F1 1.6–1.8 × as long as broad	***A. verutus* Graham**
–	Ovipositor sheaths plus postcercale 1.35–1.5 × as long as metatibia; antenna with F1 2.5 × as long as broad	**10**
10	Legs with all coxae black	***A. prolixus* LaSalle & Huang**
–	Legs with all coxae yellow	***A. fukutai* Miwa & Sonan**
11	Gaster oval to circular, not longer than head and mesosoma	**12**
–	Gaster long-oval to lanceolate, longer than head and mesosoma	**18**
12	Forewing SMV with 8 dorsal setae; propodeum with plica	***A. dryocosmi* Wu & Xu**
–	Forewing SMV with 3–5 dorsal setae; propodeum without plica	**13**
13	Mid lobe of mesoscutum without median line; gaster circular, 1.0–1.1 × as long as broad	***A. magniventer* Yang**
–	Mid lobe of mesoscutum with median line, sometimes fine; gaster circular to oval, 1.0–1.7 × as long as broad	**14**
14	Antenna with F1 4.7 × as long as broad; body drab yellow with black markings	***A. albae* Yang**
–	Antenna with F1 2.0–3.0 × as long as broad; body brown to black, sometimes with yellow markings	**15**
15	Antenna with F1 short, 1.2 × as long as broad; gaster equal in length and width	***A. brevipedicellus* Yang & Cao**
–	Antenna with F1 1.5–2.0 × as long as broad; gaster length greater than width	**16**
16	Forewing with MV 3.0 × as long as STV	***A. purpurea* (Cameron)**
–	Forewing with MV 3.8–4.0 × as long as STV	**17**
17	Forewing with PMV 0.4 × as long as STV; gaster 1.4 × as long as broad	***A. dendrolimi* Yang & Cao**
–	Forewing with PMV rudimentary; gaster 1.5–1.7 × as long as broad	***A. ceroplastae* (Girault)**
18	Body yellow, usually with brown to black markings	**19**
–	Body black, sometimes with yellow markings or body bluish green without yellow markings	**22**
19	Gaster elongated lanceolate, 4.5–5.0 × as long as broad; mid lobe of mesoscutum with median line incomplete, only anterior 1/2 visible	***A. holoxanthus* Graham**
–	Gaster lanceolate, not > 4.0 × as long as broad; mid lobe of mesoscutum with median line complete	**20**
20	Antenna with F3 quadrate; propodeum with paraspiracular carina	***A. causalis* La Salle & Wu**
–	Antenna with F3 distinctly longer than broad; propodeum without paraspiracular carina	**21**
21	Gaster 1.8 × as long as broad; forewings with MV 3.0 × as long as STV (Fig. 31)	***A. pinus* Li & Xu**
–	Gaster 2.7 × as long as broad; forewings with MV 4.0 × as long as STV	***A. crypturgus* Yang**
22	Antenna with clava quite short, 1.2–1.6 × as long as broad, obtuse apically with a downwardly curved terminal spine (Fig. 27)	***A. gratus* (Giraud)**
–	Antenna with clava > 1.8 × as long as broad, not obtuse apically, terminal spine straight	**23**
23	Antenna with pedicellus shorter than F1	**24**
–	Antenna with pedicellus as long as to longer than F1	**31**
24	Antenna with F1 3.0–3.6 × as long as broad	**25**
–	Antenna with F1 1.7–2.8 × as long as broad	**27**
25	Spur of mesotibia longer than mesobasitarsus; body black without yellow markings	***A. massonianae* Yang**
–	Spur of mesotibia not longer than mesobasitarsus; body black with yellow markings	**26**
26	Spur of mesotibia approx. as long as mesobasitarsus; gaster with last tergite 1.0 × as long as broad	***A. citrinus* (Förster)**
–	Spur of mesotibia ~1/2 the length of mesobasitarsus; gaster with last tergite 1.9 × as long as broad	***A. blastophagusi* Yang**
27	Propodeal callus with 5 setae on each side; forewings with MV 4.3 × as long as STV	***A. pontaniae* Yang**
–	Either propodeal callus with 2 setae on each side or forewings with MV 3.0–3.6 × as long as STV	**28**
28	Mid lobe of mesoscutum without median line; propodeal callus with 3–6 setae on each side	***A. westwoodii* (Fonscolombe)**
–	Mid lobe of mesoscutum with median line; propodeal callus with 2 setae on each side	**29**
29	Gaster 1.8–2.3 × as long as broad; antenna with F3 1.3–1.5 × as long as broad	***A. diversus* (Förster)**
–	Gaster 2.9–4.4 × as long as broad; antenna with F3 1.5–2.0 × as long as broad	**30**
30	Antenna with F3 2.0 × as long as broad; propodeum medially approx. as long as dorsellum	***A. csokakoensis* (Erdös)**
–	Antenna with F3 1.5–1.8 × as long as broad; propodeum medially 0.6–0.7 × as long as dorsellum	***A. torquentis* Graham**
31	Antenna with F1 extremely short, only 0.2–0.25 × as long as pedicellus	***A. ciliatus* (Nees)**
–	Antenna with F1 at most slightly shorter than pedicellus	**32**
32	Mid lobe of mesoscutum without median line; spur of mesotibia < 1/2 the length of mesobasitarsus	***A. clavicornis* (Zetterstedt)**
–	Mid lobe of mesoscutum with median line, sometimes fine; spur of mesotibia > 1/2 the length of mesobasitarsus	**33**
33	Antenna with clava 3.0–3.2 × as long as broad	**34**
–	Antenna with clava 1.8–2.8 × as long as broad	**35**
34	Gaster approx. as long as head plus mesosoma; tips of ovipositor sheaths hardly projecting (Fig. 7)	***A. ligus* (Walker)**
–	Gaster 1.4 × as long as head plus mesosoma; tips of ovipositor sheaths distinctly projecting, approx. the length of last tergite	***A. ilexi* Sheng & Zhao**
35	Gaster with longest seta of each cercus 1.5–1.6 × the length of the next longest; body usually with strong green to blue metallic tints	**36**
–	Gaster with longest seta of each cercus 2.0 × the length of the next longest; body usually with weak green to blue metallic tints	**37**
36	Propodeal callus with 4–6 setae on each side; gaster 2.0–2.5 × as long as broad (Figs 10, 11)	***A. occidentalis* Graham**
–	Propodeal callus with 2 setae on each side; gaster 2.5–4.8 × as long as broad (Fig. 21)	***A. epicharmus* (Walker)**
37	Propodeum medially approx. as long as dorsellum; dorsellum yellow (Fig. 14)	***A. viridinitens* Graham**
–	Propodeum medially shorter than dorsellum; dorsellum black	**38**
38	Ovipositor sheaths plus the length postcercale 0.6 × as long as metatibia; body length 1.2–1.4 mm (Figs 28, 29)	***A. minimus* (Ratzeburg)**
–	Ovipositor sheaths plus the length postcercale 0.7–1.1 × as long as metatibia; body length 1.4–2.1 mm (Fig. 18)	***A. caudatus* Westwood**

### ﻿Species descriptions

#### ﻿ Aprostocetus maculosus sp. nov.

Taxon classificationAnimaliaHymenopteraEulophidae

C17F1EFC-0D7F-5A1C-A2FA-096CEF679778

https://zoobank.org/7813B3B4-88CA-4149-92C6-8174ADF7F262

Figs 1–6

##### Type material.

***Holotype***, female [on card], China • Hainan Province, Hainan Limushan Forest Park, 30.IV.2021, Gang Fu, Ming-Rui Li, by sweeping. Deposited in YCTU. ***Paratypes***: • 5 females, 3 males: [2 females, 2 males on cards], same data as holotype; [1 female, 1 male on cards], • **Hainan Province**, Chengmai County, Jinjiang Town, 24.IV.2021, Gang Fu, Ming-Rui Li, by yellow-pan trapping; [2 females on slides], • **Hainan Province**, Wuzhishan City, Qianchi Village, 24.IV.2021, Gang Fu, Ming-Rui Li, by sweeping. All deposited in YCTU.

##### Diagnosis.

Mesosoma with area surrounding median line black; metasoma black with two yellow spots on 1^st^−5^th^ gastral tergites dorsally; mid lobe of mesoscutum with two rows of adnotaular setae on each side, each row with 5–6 setae. Among Chinese species of *Aprostocetus*, *A. maculosus* is similar to *A. pinus* in having a yellow body with black markings but can be separated from this species by the following combination of characters: area surrounding median line black (vs yellow); mid lobe of mesoscutum with two rows of adnotaular setae on each sided (vs one row). Among non-Chinese species, *A. maculosus* is similar to *A. narius* Narendran, 2007 in its yellow body with black markings but can be separated from this species by the following combination of characters: POL 1.7–2.0 × OOL (vs 4.5×); mandibles distinctly tridentate (vs bidentate); propodeum with median carina broad and short (vs absent).

##### Description.

**Figures 1–6.**
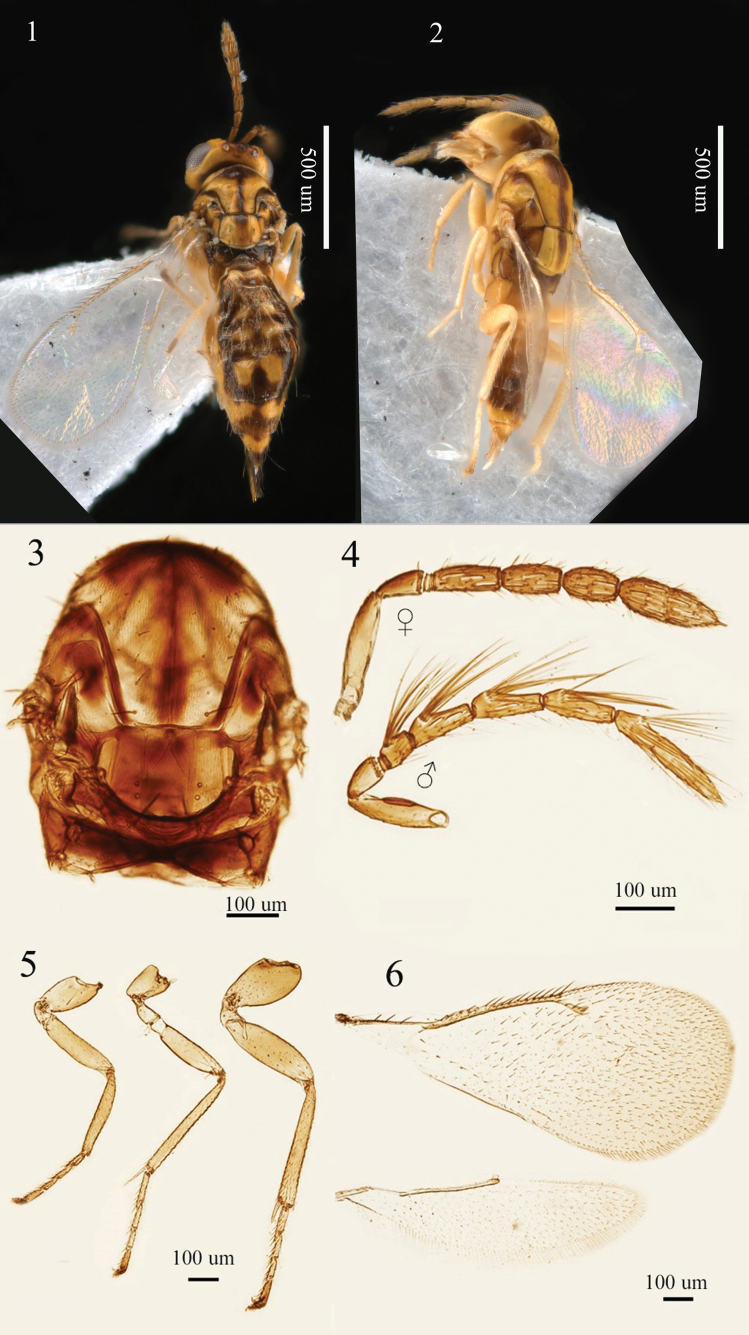
*Aprostocetus maculosus* sp. nov. 1. Holotype, female, habitus, dorsal view; 2. Paratype, male, habitus, lateral view; 3. Mesosoma, dorsal view; 4. Antenna, lateral view; 5. Legs, lateral view, from left to right: fore, mid, and hind legs; 6. Fore and hind wings, dorsal view.

**Female.** Body (Fig. 1) length 1.9–2.2 (2.2) mm. Head and mesosoma mainly yellow, scape posterior 1/3 brown on dorsal side, pedicellus and flagellum brown; area surrounding ocelli dark brown; occiput black; anterior 1/3 of pronotum black; area surrounding median line, anteromedial area of mid lobe of mesoscutum in some specimens, notaulus, anterior part of scapula and axillae black, usually reddish-brown areas around these black areas; area between submedian grooves mainly reddish-brown except anteromedial area dark brown, only anteromedial area reddish-brown in some specimens; dorsellum yellowish-white; propodeum black; legs yellow except terminal tarsomere brown; wings hyaline, veins brown. Metasoma yellow ventrally and laterally, black with two yellow spots on 1^st^−5^th^ gastral tergites dorsally, sometimes two yellow markings connected to form one yellow marking, 6^th^ and 7^th^ gastral tergites mainly yellow, only the end black; ovipositor sheaths black.

Head in dorsal view (Fig. 1), 1.8–2.2 × (1.8×) as broad as long, slightly broader than mesosoma. Frons with a median line, not depressed, without reticulation; POL 1.7–2.0 × (1.9×) OOL, OOL 1.8–2.0 × (2.0×) OD. Malar sulcus straight; malar space 0.65 × as long as eye; mouth cavity 1.3 × as wide as malar space. Anterior margin of clypeus bidentate, mandibles distinctly tridentate. Lower edge of antennal torulus above ventral edge of eyes. Antenna (Fig. 4) with scape shorter than an eye, not reaching vertex, 4.0–4.3 × (4.3×) as long as broad, pedicellus shorter than to as long as F1, 1.8–2.1 × (1.8×) as long as broad, F1 longer than F2 and F3, F1−F3: 2.0–2.5 × (2.5×), 2.0–2.1 × (2.0×), 1.6–2.0 × (1.7×) as long as broad respectively; clava slightly shorter than to as long as F2 and F3 combined, 2.8–3.1 × (3.1×) as long as broad, terminal spine shorter than C3, 0.3–0.4 × (0.4×) as long as C3, flagellum with short setae and numerous sensilla on each segment.

Mesosoma (Figs 1, 3) 1.3–1.5 × (1.4×) as long as broad. Pronotum short, arched. Mid lobe of mesoscutum 1.2 × as broad as long, median line distinct, reticulation fine, with 2 rows of adnotaular setae on each side, each row with 5–6 setae. Scutellum 1.4 × as broad as long, anterior pair of setae situated behind middle, submedian grooves and sublateral grooves distinct, distance between submedian grooves broader than distance between submedian grooves and sublateral grooves. Dorsellum partly covered by hind edge of scutellum, 4.0 × as broad as long. Propodeum medially slightly shorter than dorsellum, median carina broad and short, reticulation fine, spiracle subcircular, moderate size, almost touching hind edge of metanotum, callus with three to four setae on each side. Legs (Fig. 5) slender, spur of metatibia slightly shorter than the length of metabasitarsus. Forewing (Fig. 6) 2.0–2.4 × (2.2×) as long as broad, SMV with three to four (three) dorsal setae, costal cell relatively broad, shorter than MV, 0.8 × as long as MV; MV 3.5–4.0 × (3.9×) as long as STV; speculum closed and extending below MV.

Gastral petiole present and transverse. Gaster (Fig. 1) lanceolate, 1.8–2.0 × (1.8×) as long as head and mesosoma combined, 2.8–3.2 × (3.0×) as long as broad, longest seta of each cercus 2.0 × longer than the second longest seta. Ovipositor sheaths distinctly extending beyond tip of metasoma, approx. as long as 7^th^ gastral tergite.

**Male.** Very similar to female. Body (Fig. 2) length 1.4 mm. Metasoma sometimes with more extensive black markings (Fig. 2). Antenna with scape 3.5 × as long as broad, ventral plaque placed in the middle, 0.34 × as long as scape; pedicellus longer than F1, 1.7 × as long as broad; flagellum with numerous long setae at base of each segment, F1−F4: 1.4×, 2.2×, 2.7×, 2.7 × as long as broad respectively; clava 7.1 × as long as broad, terminal spine short; sensilla numerous, oblong. Mesosoma 1.56 × as long as broad. Forewing with costal cell slightly shorter than MV, MV 3.2 × as long as STV. Metasoma slightly longer than mesosoma, 2.4 × as long as broad.

##### Host.

Unknown.

##### Distribution.

China (Guangdong, Hainan, Guangxi).

##### Etymology.

The epithet *maculosus* refers to the many spots of the gaster dorsally.

#### ﻿ Aprostocetus ligus

Taxon classificationAnimaliaHymenopteraEulophidae

(Walker, 1839)

4C82A7B5-7A99-566C-930D-D946D6D204AC

Figs 7–9

Cirrospilus ligus Walker, 1839a: 300.
Cirrospilus oxathres Walker, 1839a: 299. [Synonymized by Graham 1987: 335]
Tetrastichus ligus : Walker 1846: 76.
Tetrastichus oxathres : Walker 1848: 148.
Aprostocetus oxathres : Graham 1961: 60.
Aprostocetus ligus : Graham 1961: 60.

##### Material examined.

• 2 females: [2 females on cards], China, Chongqin City, Simianshan Mountain, 2.VII.2018, Guang-Xin Wang, Jun-Jie Fan, by sweeping, deposited in YCTU.

##### Diagnosis.

**Figures 7–9.**
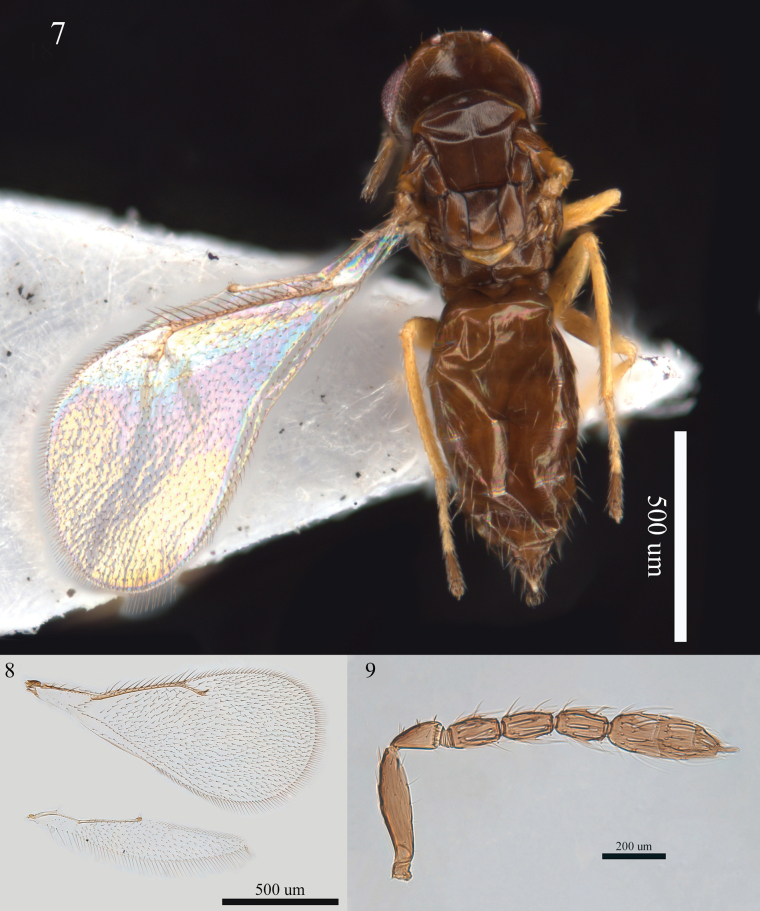
*Aprostocetus ligus* (Walker), female. 7. Habitus, dorsal view; 8. Fore and hind wings, dorsal view; 9. Antenna, lateral view.

Female. Antenna (Fig. 9) with F1 1.7×, F2 2.0×, F3 1.8 × as long as broad, clava 3.2 × as long as broad with terminal spine nearly as long as C3. Thorax with very superficial reticulation, median line of mid lobe of mesoscutum fine but distinct, four adnotaular setae on each side; propodeum medially as long as dorsellum; callus with two setae. Forewing (Fig. 8) with three or four dorsal setae, MV 3.4 × STV. Gaster (Fig. 7) ovate, 1.7 × as long as broad, nearly as long as head plus thorax, tips of ovipositor sheaths hardly projecting, longest seta of each cercus nearly 2.0 × as long as next longest. Body black without metallic reflection.

Male. Unknown for Chinese material.

##### Hosts.

Unknown.

##### Distribution.

China (Chongqin), England (Graham 1987), Sweden (Hedqvist 2003), Russia (Yegorenkova et al. 2007).

#### ﻿ Aprostocetus occidentalis

Taxon classificationAnimaliaHymenopteraEulophidae

Graham, 1987

89E4AAFC-C790-5380-9F35-74C0A3B71A59

Figs 10–13

Aprostocetus occidentalis Graham, 1987: 266.

##### Material examined.

• 2 females: [2 females on cards], China, Xizang Autonomous Region, Jilong Town, Jilong Village, 30. IV.2021, Jun-Jie Fan, Ju Wu, by sweeping, deposited in YCTU.

##### Diagnosis.

**Figures 10–13.**
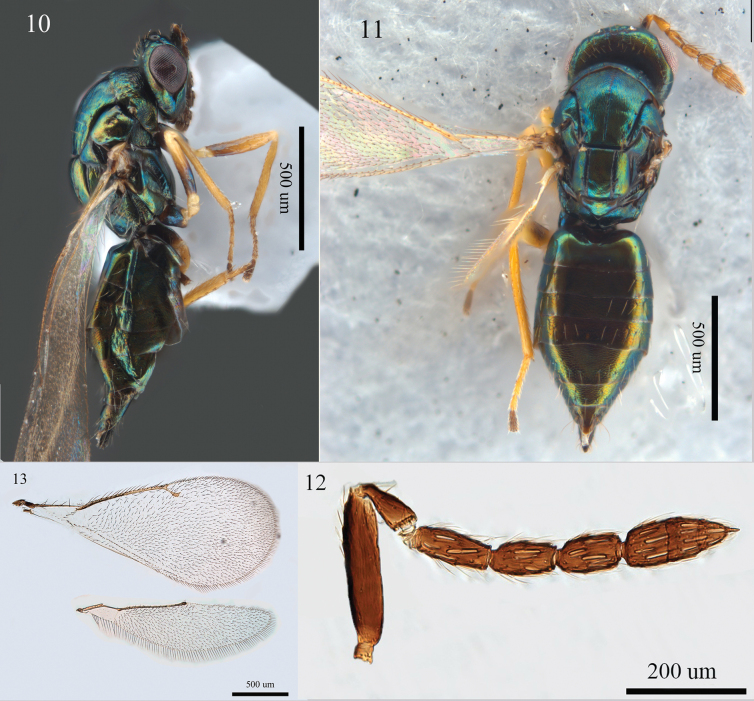
*Aprostocetus occidentalis* Graham, female. 10. Habitus, dorsal view; 11. Habitus, lateral view; 12. Antenna, lateral view; 13. Fore and hind wings, dorsal view.

Female. Malar sulcus (Fig. 10) with a subtriangular fovea below eyes. Antenna (Fig. 12) with F1 2.2–2.3×, F2 1.9–2.0×, F3 1.7 × as long as broad, clava 2.5–2.7 × as long as broad with terminal spine 0.5 × as long as C3. Thorax (Figs 10, 11) with superficial, hardly raised reticulation, median line of mid lobe of mesoscutum distinct, three to five adnotaular setae on each side; propodeum callus with five to six setae. Forewing (Fig. 13) SMV with five dorsal setae, MV 4.0 × as long as STV. Gaster lanceolate, 2.0–2.5 × as long as broad, with superficial reticulation. Body (Figs 10, 11) bright green with distinct bronze reflection. Antennal scape, all coxae and femora mainly concolorous with body.

Male. Unknown for Chinese material.

##### Hosts.

Unknown.

##### Distribution.

China (Xizang [new record]), Italy, Spain (Graham 1987), Madeira (Koponen and Askew 2002), Russia (Yegorenkova et al. 2007).

##### Comments.

Graham (1987) reported that *A. occidentalis* has the terminal spine as long as C3; however, the specimens we examined have the terminal spine only 0.5 × as long as C3.

#### ﻿ Aprostocetus viridinitens

Taxon classificationAnimaliaHymenopteraEulophidae

Graham, 1987

40B110AC-F447-512C-9C8A-87E0024D9FDD

Figs 14–17

Aprostocetus viridinitens Graham, 1987: 345.

##### Material examined.

• 2 females: [2 females on cards], China, Inner Mongolia Autonomous Region, Hohhot City, Hulun Lake, 7–8. VII.2021, Yuan-Yuan Jin, Yue Qin, by yellow pan trapping, deposited in YCTU.

##### Diagnosis.

**Figures 14–17.**
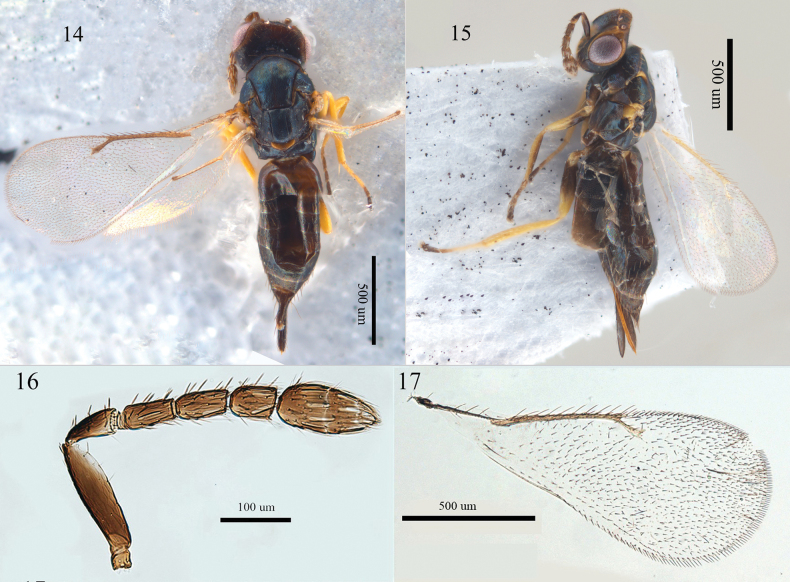
*Aprostocetus viridinitens* Graham, female. 14. Habitus, dorsal view; 15. Habitus, lateral view; 16. Antenna, lateral view; 17. Forewing, dorsal view.

Female. Head and thorax metallic blue except eye margin, tegulae and dorsellum yellow (Figs 14, 15). Antennal scape and pedicellus black (Fig. 16), all coxae and femora mainly black. Antenna (Fig. 16) with F1 1.9 × as long as broad, clava 2.3 × as long as broad with terminal spine inconspicuous. Thorax with superficial, hardly raised reticulation, median line of mid lobe of mesoscutum weak, four to five adnotaular setae on each side; propodeum callus with two setae. Gaster acute, 3.0 × as long as broad, 1.8 × as long as thorax.

Male. Unknown for Chinese material.

##### Hosts.

Unknown.

##### Distribution.

China (Inner Mongolia [new record]), Czech Republic, France, England (Graham 1987), Sweden (Hedqvist 2003), Russia (Yefremova 2002).

#### ﻿ Aprostocetus caudatus

Taxon classificationAnimaliaHymenopteraEulophidae

Westwood, 1833

B3F8AD6A-B662-5698-9D86-560AABF8AC1D

Figs 18–20

Aprostocetus caudatus Westwood, 1833: 444.
Cirrospilus phalis Walker, 1839c: 418. [Synonymized by Graham 1987: 236]
Cirrospilus mutilia Walker, 1839a: 322. [Synonymized by Graham 1961: 52]
Cirrospilus trabea Walker, 1839a: 323. [Synonymized by Graham 1961: 52]
Tetrastichus crassicauda Thomson, 1878: 293. [Synonymized by Graham 1961: 52]
Eulophus tristis Nees in Esenbeck, 1834: 188. [Synonymized by Dalla 1898: 2]
Tetrastichus caudatus : Walker 1846: 78.
Tetrastichus phalis : Walker 1846: 78.
Tetrastichus mutilia : Walker 1848: 150.
Tetrastichus trabea : Walker 1848: 150.
Aprostocetus crassicauda : Erdös 1956: 43.

##### Material examined.

• 1 female: [1 female on card], China, Neimenggu Province, Duolun County, Shanhou Village, 28.VII.2023, Yuan-Yuan Jin, by sweeping, deposited in YCTU.

##### Diagnosis.

**Figures 18–20.**
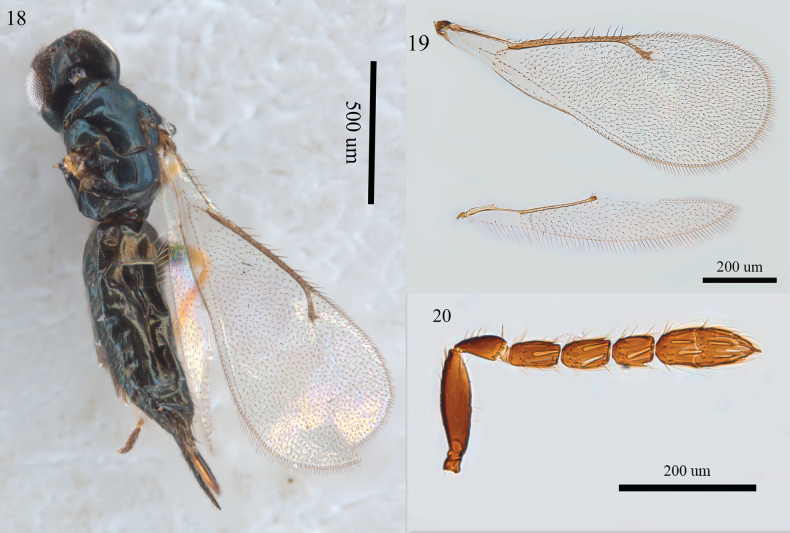
*Aprostocetus caudatus* Westwood, female. 18. Habitus, dorsal view; 19. Fore and hind wings, dorsal view; 20. Antenna, lateral view.

Female. Antenna (Fig. 20) with F1 1.8×, F2 1.7×, F3 1.4 × as long as broad, clava 2.6 × as long as broad with terminal spine 0.5 × as long as C3. Thorax (Fig. 18) with superficial, hardly raised reticulation, median line of mid lobe of mesoscutum fine, three adnotaular setae on each side; propodeum callus with two setae. Forewing SMV with four dorsal setae, MV 3.7 × STV. Gaster (Fig. 18) lanceolate, 3.2 × as long as broad, with superficial reticulation, last gastral tergite 2.0 × as long as broad, projecting part of ovipositor approx. as long as postcercale. Body black with violet metallic reflection.

Male. Unknown for Chinese material.

##### Hosts.

Unknown from China. Non-Chinese records include *Dasineura alopecuri* (Reuter, 1895) (Diptera: Cecidomyiidae) (Thompson 1955).

##### Distribution.

China (Hainan [new record], Guangxi [Zhu and Huang 2001]), Austria, Czech Republic, Slovakia, Germany, France, England, Greece, Hungary, Ireland, Italy, Netherlands, Sweden (Graham 1987), Turkey (Sakaltas and Gençer 2005), Russia (Yegorenkova et al. 2007).

#### ﻿ Aprostocetus epicharmus

Taxon classificationAnimaliaHymenopteraEulophidae

(Walker, 1839)

D638AA8F-D58D-5755-81C3-1E34EA40F313

Figs 21–23

Cirrospilus epicharmus Walker, 1839d: 180.
Cirrospilus vincius Walker, 1839a: 317. [Synonymized by Graham 1987: 222].
Tetrastichus epicharmus : Walker 1846: 74.
Tetrastichus variegatus Szelényi, 1941: 406. [Synonymized by Domenichini 1966: 154].
Geniocerus variegatus : Erdös 1956: 46.
Aprostocetus vincius : Graham 1961: 52.
Aprostocetus epicharmus : Graham 1961: 52.

##### Material examined.

• 2 females: [2 females on cards], China, Inner Mongolia Autonomous Region, Xilingol City, Duolun County, 28.VII.2023, Yuan-Yuan Jin, by sweeping, deposited in YCTU.

##### Diagnosis.

**Figures 21–23.**
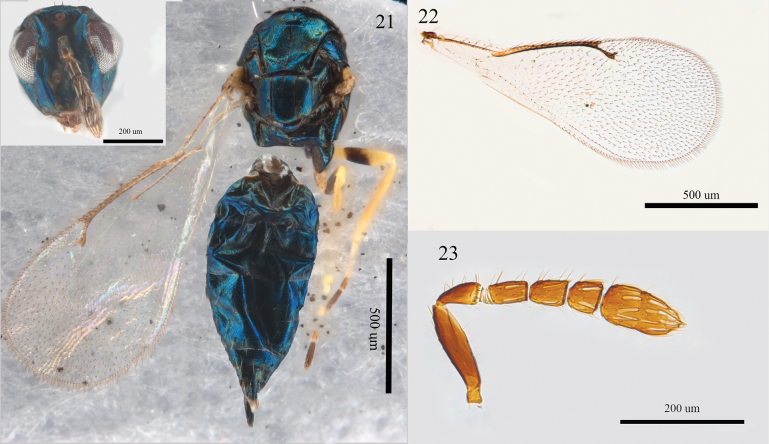
*Aprostocetus epicharmus* (Walker), female. 21. Habitus, dorsal view; 22. Forewing, dorsal view; 23. Antenna, lateral view.

Female. Antenna (Fig. 23) with F1 slightly shorter than pedicellus, 1.8 × as long as broad, F2 1.5 × as long as broad, F3 quadrate; clava broader than F3, 2.1 × as long as broad, terminal spine 0.5 × as long as C3. Forewing (Fig. 22), 2.3 × as long as broad; MV 3.7–3.8 × STV; SMV with four dorsal setae, PMV developed, only 0.2 × ST × STV. Body (Fig. 21) black with blue metallic tint.

Male. Unknown for Chinese material.

##### Hosts.

Unknown from China. Non-Chinese records include *Contarinia medicaginis* Kieffer, 1895, *Jaapiella medicaginis* (Rübsaamen, 1912), *Dasineura brassicae* (Winnertz, 1853), *D. gleditchiae* (Osten Sacken, 1866) *D. papaveris* (Winnertz, 1853) (Diptera: Cecidomyiidae) (Graham 1987), *Aylax minor* Hartig, 1840, *A. papaveris* (Perris, 1839), *Isocolus scabiosae* (Giraud, 1859), *Phanacis centaureae* Förster, 1860 (Hymenoptera: Cynipidae) (Askew et al. 2006).

##### Distribution.

China (Inner Mongolia [new record], Gansu [Zhang et al. 2007]), Czech Republic, France (Bouček 1966), Netherlands (Gijswijt 2003), Poland (Czajkowska 1978), Spain (Askew et al. 2006), Germany, Greece, Hungary, Ireland, Italy, Montenegro, Sweden, England (Graham 1987), Russia (Yefremova 2002).

#### ﻿ Aprostocetus gratus

Taxon classificationAnimaliaHymenopteraEulophidae

(Giraud, 1863)

8AC9C7E7-1174-58E0-9425-DFABAAB1B4EA

Figs 24–27

Tetrastichus gratus Giraud, 1863: 1275.
Tetrastichus deplanatus Thomson, 1878: 291. [Synonymized by Graham 1961: 49]
Tetrastichus thomsonii Dalla Torre, 1898: 23. [Synonymized by Graham 1961: 49]
Geniocerus gratus : Erdös 1956: 51.
Aprostocetus gratus : Graham 1961: 49.
Tetrastichus badulini Kostjukov, 1977: 191. [Synonymized by Graham 1987: 283]

##### Material examined.

• 2 females: [2 females on cards], China, Jiangsu Province, NanTong City, Coastal Levee, 4.VI.2023, Wen-Jian Li, by sweeping, deposited in YCTU.

##### Diagnosis.

**Figures 24–27.**
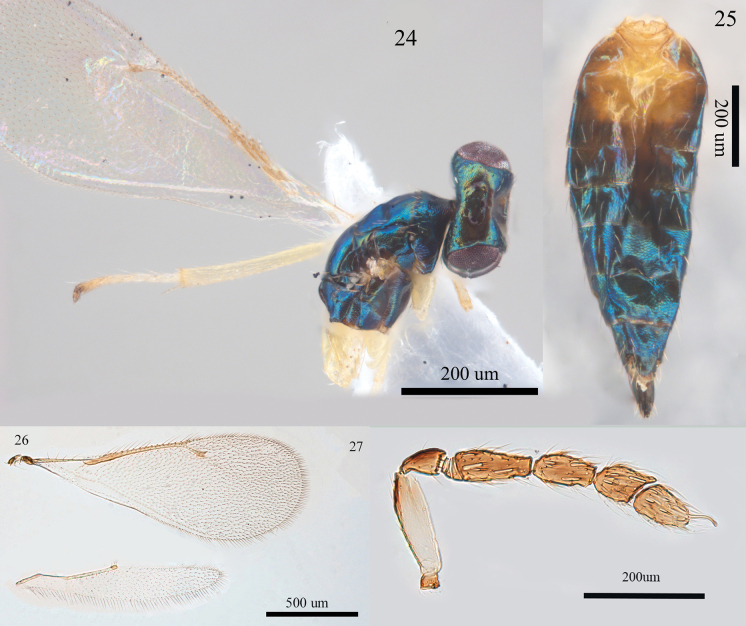
*Aprostocetus gratus* (Giraud), female; 24. Habitus of head and mesosoma, lateral view; 25. Habitus of metasoma, dorsal view; 26. Fore and hind wings, dorsal view; 27. Antenna, lateral view.

Female. Antenna (Fig. 27) with F1 distinctly longer than F2, 2.5 × as long as broad, F3 apical margin oblique, dorsal edge distinctly longer than ventral edge, clava quite short, solid, obtuse apically, terminal spine downward curved and slender, ~0.5 × as long as the clava itself, apical seta of spine rudimentary. Forewing (Fig. 26) narrow, 2.5–2.6 × as long as broad; MV 3.9–4.0 × STV; SMV with four dorsal setae; speculum very narrow. Body (Figs 24, 25) black with bright blue metallic tint; legs yellowish with terminal tarsus brown; gaster basal 1/4 yellow.

Male. Unknown for Chinese material.

##### Hosts.

Unknown from China. Non-Chinese records include *Giraudiella inclusa* (Frauenfeld, 1862), *Lasioptera arundinis* Schiner, 1854 (Diptera: Cecidomyiidae) (Graham 1987).

##### Distribution.

China (Jiangsu [new record], Gansu [Zhang et al. 2007]), Moldova (Bouček 1966), Romania (Tudor and Roman 1973), Austria, Netherlands, former Czechoslovakia, Denmark, Germany, Finland, France, Ireland, Hungary, Italy, Sweden, United Kingdom, Russia (Graham 1987).

##### Comments.

This species can be distinguished from other *Aprostocetus* species by the unique F3 and clava.

#### ﻿ Aprostocetus minimus

Taxon classificationAnimaliaHymenopteraEulophidae

(Ratzeburg, 1848)

89F5E9FF-6963-5CC8-A8CA-DF0DBA5DD705

Figs 28, 29

Geniocerus minimus Ratzeburg, 1848: 175.
Tetrastichus minimus : Dalla 1898: 598.

##### Material examined.

2 females: [2 females on cards], China, Heilongjiang Province, Hegang City, Wuzhishan Park, 22. VII.2020, Ming-Rui Li, by sweeping, deposited in YCTU.

##### Diagnosis.

**Figures 28, 29.**
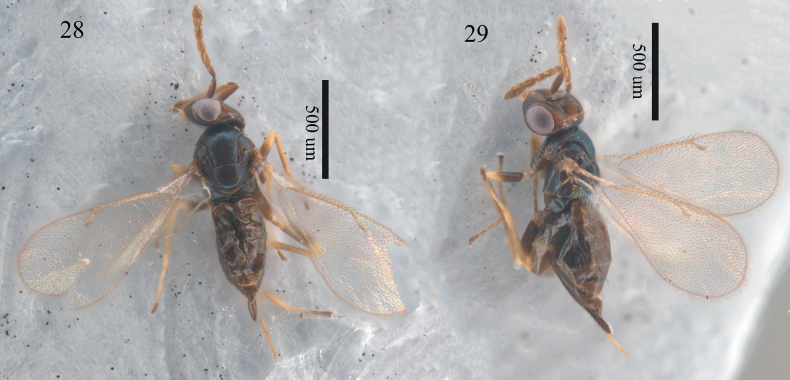
*Aprostocetus minimus* (Ratzeburg), female. 28. Habitus, dorsal view; 29. Habitus, lateral view.

Female. Body small (Figs 28, 29), length 1.3 mm. Thorax with superficial reticulation, median line of mid lobe of mesoscutum distinct; mid lobe of mesoscutum with three to four adnotaular setae on each side; propodeum medially shorter than dorsellum. Forewing with four dorsal setae on SMV, speculum small, MV 3.5 × STV. Body black, head and thorax with bluish metallic tint.

##### Hosts.

Unknown from China. Non-Chinese records include *Rabdophaga nervorum* (Kieffer, 1895), *R. salicis* (Schrank, 1803), and *R. rosaria* (Loew, 1850) (Diptera: Cecidomyiidae) (Domenichini 1966).

##### Distribution.

China (Heilongjiang [new record], Gansu), Germany, Netherlands (Graham 1987), Sweden (Hedqvist 2003), Russia (Yegorenkova et al. 2007).

#### ﻿ Aprostocetus microcosmus

Taxon classificationAnimaliaHymenopteraEulophidae

(Girault, 1917)

98476408-927C-5B7D-8626-7EBE77D109E9

Fig. 30

Aprostocetus granulatus Ashmead, 1888: 105. [Synonymized by Burk 1975: 142].
Tetrastichus microcosmus Girault, 1917: 22.
Tetrastichus asperulus Graham, 1981: 2. [Synonymized by Graham and LaSalle 1991: 91]
Aprostocetus asperulus : Graham 1987: 212.
Aprostocetus granulatus : LaSalle 1994: 144.
Aprostocetus microcosmus : Zhu and Huang 2002: 598.

##### Material examined.

9 females: [2 females on cards], China • Hainan Province, Qiongzhong County, Limushan Forest Park, 30. IV.2021, Ming-Rui Li, Gang Fu, by sweeping; [3 females on cards], China • Hainan Province, Wuzhishan City, Qianchi Village, 10. V.2021, Ming-Rui Li, Gang Fu, by sweeping; [2 females on cards], China • Hainan Province, Danzhou City, Lianhuashan Forest Park, 28. IV.2021, Ming-Rui Li, Gang Fu, by sweeping; [2 females on cards], China • Yunnan Province, Chuxiong City, Konglongshan Town, 3. VII.2022, Ren-Tao Xu, Ke-Yi Wang, by sweeping; deposited in YCTU.

##### Diagnosis.

**Figure 30.**
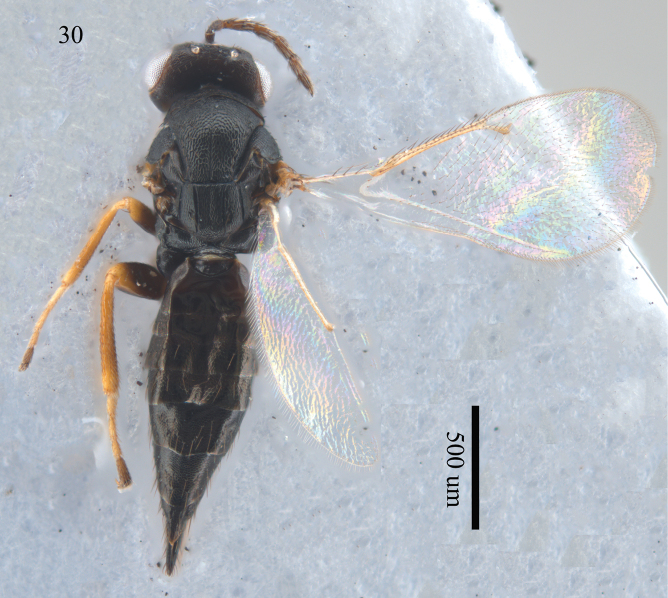
*Aprostocetus microcosmus* (Girault), female habitus, dorsal view.

Female. Thorax (Fig. 30) with dull, distinctly raised reticulation, median line of mid lobe of mesoscutum distinct but disappearing anteriorly; areoles of reticulation are mostly a little longer than broad; mid lobe of mesoscutum with 2 rows of adnotaular setae; callus with three to four setae. Forewing SMV with three to four dorsal setae, speculum open below, extending nearly to STV. Gaster (Fig. 30) lanceolate, strongly acute, 1.5 × as long as thorax plus head, with superficial scaly reticulation. Body black, without metallic luster.

##### Hosts.

Unknown.

##### Distribution.

China (Hainan, Yunnan [new records], Guangxi [Zhu and Huang 2002]), Canary Islands, Cape Verde Islands, India, Madeira, Trinidad & Tobago (Graham 1987), Australia (Bouček 1988).

##### Comments.

According to UCD Community (2023), its distribution in China includes Guangxi and Shanxi. However, according Zhu and Huang (2002), its distribution is Shangsi County, Guangxi Province. This could be a confusion between “Shanxi” and “Shangsi”.

#### ﻿ Aprostocetus pinus

Taxon classificationAnimaliaHymenopteraEulophidae

Li & Xu, 2014

29D9F83F-8459-5BA4-A3CB-C2999554FD6B

Fig. 31

Aprostocetus pinus Li & Xu, in Li et al. 2014: 393.

##### Material examined.

• 4 females on cards, China, Jiangsu Province, Yancheng City, Xinyang Port Sluice, 29. VIII.2023, Wen-Jian Li, Yong-Qiang Zhao, by sweeping, deposited in YCTU.

##### Diagnosis.

**Figure 31.**
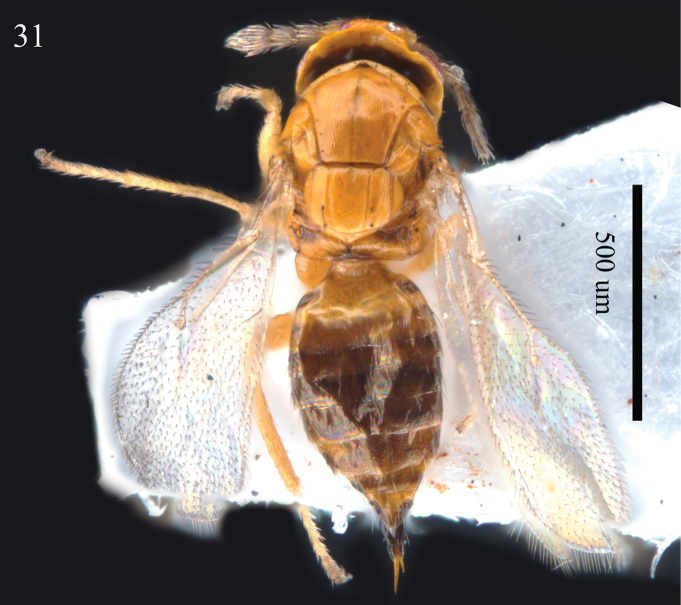
*Aprostocetus pinus* Li & Xu, female habitus, dorsal view.

Female. Thorax (Fig. 31) with superficial reticulation, median line of mid lobe of mesoscutum complete but weak; areoles of reticulation distinctly longer than broad; mid lobe of mesoscutum with five adnotaular setae on each side; propodeum carina thin and forked, forming a cup-shaped anterior-medially, callus with two setae. Forewing with four dorsal setae on SMV, speculum small, MV 3.0 × STV. Head yellow with occiput dark brown, thorax yellow with propodeum partly or mainly dark brown, gaster mainly black.

##### Hosts.

*Pinus massoniana* (Pinaceae) (Li et al. 2014).

##### Distribution.

China (Jiangsu [new record], Zhejiang).

##### Comments.

The specimens we collected have four dorsal setae on the SMV compared to five dorsal setae in the original description (Li et al. 2014).

#### ﻿ Aprostocetus nigroplaque

Taxon classificationAnimaliaHymenopteraEulophidae

Yang & Cao, 2015

1D56DC06-7674-5064-B234-97CC4B168041

Figs 32–34

Aprostocetus nigroplaque Yang & Cao in Yang and Cao 2015: 88.

##### Material examined.

• 2 females on cards, China, Tianjin City, Tianjin Academy of Agricultural Sciences, 13–30.VIII.2022, Guo-Hao Zu, by Malaise trapping, deposited in YCTU.

##### Diagnosis.

**Figures 32–34.**
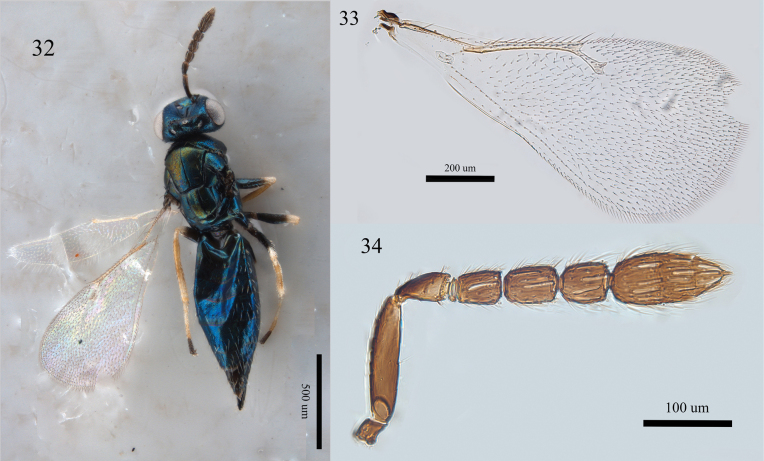
*Aprostocetus nigroplaque* Yang & Cao, female; 32. Habitus, dorsal view; 33. Forewing, dorsal view; 34. Antenna, lateral view.

Female. Median line of mid lobe of mesoscutum complete and distinct (Fig. 32); mid lobe of mesoscutum with five adnotaular setae on each side; propodeum carina broad, medially slightly shorter than dorsellum, callus with four setae. Forewing (Fig. 33) with two dorsal setae on SMV, costal cell longer than MV, MV short, 2.0 × STV. Gaster long-oval, 2.5 × as long as broad. Body (Fig. 32) metallic blue-green; antennae, scape, all coxae and femora mainly concolorous with body.

##### Hosts.

*Epinotia nisella* (Clerck, 1759) (Lepidoptera: Tortricidae) (Yang and Cao 2015).

##### Distribution.

China (Tianjin [new record], Shandong (Yang and Cao 2015)).

### ﻿Key to species of the subgenus Ootetrastichus Perkins from China (females)

**Table:** 

1	Body yellow with red and black markings	***A. muiri* (Perkins)**
–	Body mainly black with green to blue metallic tints	**2**
2	Ovipositor sheaths far exserted, their projecting part usually longer than the whole body (Fig. 35); SMV with 3–5 dorsal setae……………	***A. percaudatus* (Silvestri)**
–	Ovipositor sheaths less exserted, their projecting part ≤ 1/2the length of the gaster (e.g. Fig. 41); SMV with 2 dorsal setae	**3**
3	Antenna with F1 at least 6.0 × as long as broad	4
–	Antenna with F1 3.0–5.0 × as long as broad	**5**
4	Antenna with F1 at least 8.0 × as long as broad, F3 at least 4.0 × as long as broad; callus with 4 setae	***A. leptocerus* (Graham)**
–	Antenna with F1 6.0 × as long as broad, F3 3.0 × as long as broad; callus with 2 setae	***A. eupatorii* Kurdjumov**
5	Forewing broad, 2.1–2.2 × as long as broad	***A. mycerinus* (Walker)**
–	Forewing narrow, 2.5–3.5 × as long as broad	**6**
6	Antenna with F1 4.0 × as long as broad	***A. formosanus* (Timberlake)**
–	Antenna with F1 1.7–3.0 × as long as broad	**7**
7	Antenna with clava 3.5–4.0 × as long as broad	***A. mandanis* (Walker)**
–	Antenna with clava 3.0 × as long as broad	***A. crino* (Walker)**

#### ﻿ Aprostocetus percaudatus

Taxon classificationAnimaliaHymenopteraEulophidae

(Silvestri, 1920)

82DB6E8C-DC62-50BF-9455-F35B2AA69DB6

Figs 35–37

Tetrastichus percaudatus Silvestri, 1920: 241.
Terebratella indica Shafee & Rizvi, 1985: 377. [Synonymized by Hayat and Shahi 2004: 307.]
Aprostocetus (Ootetrastichus) percaudatus : Graham 1987: 112.

##### Material examined.

3 females: [1 female on card], China • Shandong Province, Qingdao City, Mashan Mountain, 16.VII.2014, Guo-Hao Zu, Si-Zhu Liu, Zhi-Guang Wu, Hui Geng, by sweeping; [2 females on cards], China • Yunnan Province, Shuangjiang County, 21.IV.2013, Guo-Hao Zu, Xiang-Xiang Jin, Chao Zhang, by sweeping. All deposited in YCTU.

##### Diagnosis.

**Figures 35–37.**
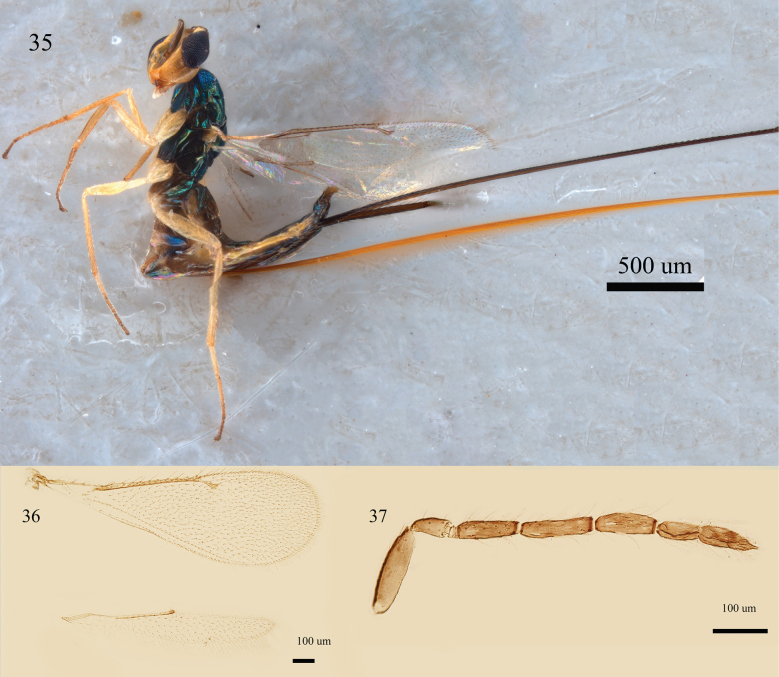
*Aprostocetus percaudatus* (Silvestri), female. 35. Habitus, dorsal view; 36. Fore and hind wings, dorsal view; 37. Antenna, lateral view.

Female. Body (Fig. 35) long, length 1.7–2.0 mm (projecting part of ovipositor sheaths not included), projecting part of ovipositor sheaths ≤ 3.0 mm in length. Body black with bright green to blue-green metallic tints.

##### Hosts.

Unknown from China. Non-Chinese records include *Oecanthus pellucens* (Scopoli, 1763) (Orthoptera: Gryllidae) (Graham 1987).

##### Distribution.

China (Shandong, Yunnan [new records]), Bulgaria, Czech Republic, Slovakia, France, Italy, Spain (Boyadzhiev 1997), Germany (Vidal 2001), Croatia, Serbia, Montenegro (Bouček 1977), Romania (Hansson 2016), Ukraine, Moldova (Bouček 1966), India (Narendran 2007).

##### Comments.

This species can be distinguished by its extremely long ovipositor sheaths. The longest projecting part of the ovipositor sheaths of one specimen was 3.0 mm.

#### ﻿ Aprostocetus crino

Taxon classificationAnimaliaHymenopteraEulophidae

(Walker, 1838)

BE7B7654-94B9-5873-BB99-400BCB84FE79

Figs 38–40

Cirrospilus crino Walker, 1838: 382.
Tetrastichus (Geniocerus) dispar Silvestri, 1920: 219. [Synonymized by Erdös 1955: 298]
Tetrastichus oecanthivorus Gahan, 1932: 743. [Synonymized by Graham 1987: 109]
Pachyscapus crino : Erdös 1954: 364.
Tetrastichus dubius Bakkendorf, 1955: 152. [Synonymized by Bouček 1961: 23]
Tetrastichus crino : Bouček 1961: 23.
Aprostocetus (Ootetrastichus) crino : Graham 1987: 109.

##### Material examined.

• 2 females on cards, China, Liaoning Province, Tieling City Fenshuiling Mountain, 24.VII.2023, Yuan-Yuan Jin, Ting-Ting Zhao, by sweeping. All deposited in YCTU.

##### Diagnosis.

**Figures 38–40.**
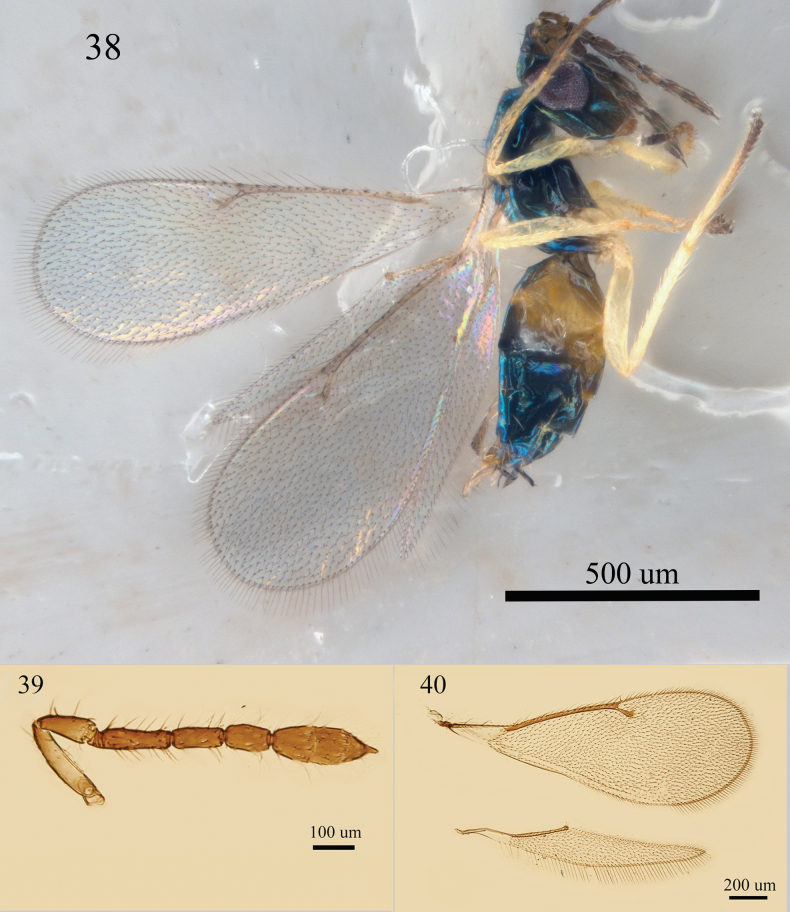
*Aprostocetus crino* (Walker), female. 38. Habitus, lateral view; 39. Antenna, lateral view; 40. Fore and hind wings, dorsal view.

Female. Antenna (Fig. 39) with F1 distinctly longer than F2, 2.5–3.0 × as long as broad, F2 2.0 × as long as broad, F3 1.8 × as long as broad, clava 3.0 × as long as broad. Forewing (Fig. 40) narrow, 2.6–2.7 × as long as broad; MV 4.5 × STV; SMV with two dorsal setae; speculum absent. Body (Fig. 38) black with bright blue metallic tint.

##### Hosts.

Unknown from China. Non-Chinese records include *Yponomeuta padellus* (Linnaeus, 1758) (Lepidoptera: Yponomeutidae) (Yegorenkova et al. 2007), *Oecanthus quadripunctatus* Beutenmueller, 1894, *O. pellucens* (Scopoli, 1763), *O. nigricornis* Walker, 1869 (Orthoptera: Gryllidae) (Graham 1987).

##### Distribution.

China (Hebei, Zhejiang, Liaoning [new record]), Czech Republic, Slovakia (Kalina 1989), Croatia, Montenegro (Bouček 1977), Andorra, France, Denmark, England, Hungary, Iceland, Ireland, Italy, Sweden, USA (Graham 1987), Bulgaria (Boyadzhiev 1997), Romania (Hansson 2016), Turkey (Sakaltas and Gençer 2005), Moldova (Bouček 1961), Netherlands (Gijswijt 2003) Germany (Vidal 2001), Russia (Yegorenkova et al. 2007).

#### ﻿ Aprostocetus mandanis

Taxon classificationAnimaliaHymenopteraEulophidae

(Walker, 1839)

64F84AA9-84E1-504A-A5FC-587440CA1F28

Figs 41, 42

Cirrospilus mandanis Walker, 1839b: 202.
Tetrastichus mandanis : Walker 1846: 74.
Anellaria conomeli Bakkendorf, 1934: 9. [Synonymized by Graham 1961: 44.]
Tetrastichus conomeli : Bakkendorf 1953: 564.
Aprostocetus (Ootetrastichus) mandanis : Graham 1987: 108.

##### Material examined.

• 2 females: [1 female on card], China • Yunnan Province, Yuanjiang County, 26–28.XI.2022, Jun-Jie Fan, Ting-Ting Zhao, Jun Wu, by sweeping; [1 female on card], China • Xizang Autonomous Region, Motuo County, Gedang Township, 31.V–5.VI.2021, Jun-Jie Fan, Jun Wu, by yellow-pan trapping. All deposited in YCTU.

##### Diagnosis.

**Figures 41, 42.**
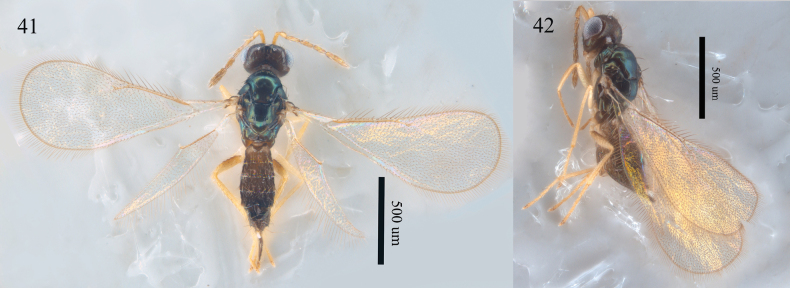
*Aprostocetus mandanis* (Walker), female. 41. Habitus, dorsal view; 42. Habitus, lateral view.

Female. Antenna (Figs 41, 42) with calva 3.5–4.0 × as long as broad, as long as F2 and F3 combined. Forewing (Figs 41, 42) narrow, 2.5–2.7 × as long as broad; MV 4.0 × STV; SMV with two dorsal setae; speculum absent. Body (Figs 41, 42) black with bright blue metallic tint.

##### Hosts.

Unknown from China. Non-Chinese records include *Conomelus anceps* (Germar, 1821) (Rothschild 1966), *C. dehneli* Fieber, 1866 (Arzone 1978), *Euconomelus Lepidus* (Boheman, 1847) (Graham 1987) (Hemiptera: Delphacidae).

##### Distribution.

China (Shanghai, Yunnan, Xizang [new record]), Bulgaria (Boyadzhiev 2003), Czech Republic, Slovakia (Kalina 1989), Denmark, United Kingdom, Hungary, Portugal, Sweden (Graham 1987), Germany (Vidal 2001), Italy (Arzone 1978), Netherlands (Gijswijt 2003).

## Supplementary Material

XML Treatment for Aprostocetus maculosus

XML Treatment for Aprostocetus ligus

XML Treatment for Aprostocetus occidentalis

XML Treatment for Aprostocetus viridinitens

XML Treatment for Aprostocetus caudatus

XML Treatment for Aprostocetus epicharmus

XML Treatment for Aprostocetus gratus

XML Treatment for Aprostocetus minimus

XML Treatment for Aprostocetus microcosmus

XML Treatment for Aprostocetus pinus

XML Treatment for Aprostocetus nigroplaque

XML Treatment for Aprostocetus percaudatus

XML Treatment for Aprostocetus crino

XML Treatment for Aprostocetus mandanis

## ﻿Additional information

### Use of AI

No use of AI was reported.

### Author ORCIDs

Wen-Jian Li https://orcid.org/0009-0004-9790-3608

Zhi-Peng Chen https://orcid.org/0009-0006-9947-5074

Guo-Hao Zu https://orcid.org/0000-0002-9892-2171

### Data availability

All of the data that support the findings of this study are available in the main text.
